# Platelet Serotonin Transporter Function Predicts Default-Mode Network Activity

**DOI:** 10.1371/journal.pone.0092543

**Published:** 2014-03-25

**Authors:** Christian Scharinger, Ulrich Rabl, Christian H. Kasess, Bernhard M. Meyer, Tina Hofmaier, Kersten Diers, Lucie Bartova, Gerald Pail, Wolfgang Huf, Zeljko Uzelac, Beate Hartinger, Klaudius Kalcher, Thomas Perkmann, Helmuth Haslacher, Andreas Meyer-Lindenberg, Siegfried Kasper, Michael Freissmuth, Christian Windischberger, Matthäus Willeit, Rupert Lanzenberger, Harald Esterbauer, Burkhard Brocke, Ewald Moser, Harald H. Sitte, Lukas Pezawas

**Affiliations:** 1 Department of Psychiatry and Psychotherapy, Medical University of Vienna, Vienna, Austria; 2 Department of Laboratory Medicine, Medical University of Vienna, Vienna, Austria; 3 Center for Medical Physics and Biomedical Engineering, Medical University of Vienna, Vienna, Austria; 4 Center for Biomolecular Medicine and Pharmacology, Medical University of Vienna, Vienna, Austria; 5 Department of Psychology, Dresden University of Technology, Dresden, Germany; 6 Department of Statistics and Probability Theory, Vienna University of Technology, Vienna, Austria; 7 Central Institute of Mental Health, University of Heidelberg, Mannheim, Germany; Universitätsklinikum Carl Gustav Carus an der Technischen Universität Dresden, Germany

## Abstract

**Background:**

The serotonin transporter (5-HTT) is abundantly expressed in humans by the serotonin transporter gene *SLC6A4* and removes serotonin (5-HT) from extracellular space. A blood-brain relationship between platelet and synaptosomal 5-HT reuptake has been suggested, but it is unknown today, if platelet 5-HT uptake can predict neural activation of human brain networks that are known to be under serotonergic influence.

**Methods:**

A functional magnetic resonance study was performed in 48 healthy subjects and maximal 5-HT uptake velocity (V_max_) was assessed in blood platelets. We used a mixed-effects multilevel analysis technique (MEMA) to test for linear relationships between whole-brain, blood-oxygen-level dependent (BOLD) activity and platelet V_max_.

**Results:**

The present study demonstrates that increases in platelet V_max_ significantly predict default-mode network (DMN) suppression in healthy subjects independent of genetic variation within *SLC6A4*. Furthermore, functional connectivity analyses indicate that platelet V_max_ is related to global DMN activation and not intrinsic DMN connectivity.

**Conclusion:**

This study provides evidence that platelet V_max_ predicts global DMN activation changes in healthy subjects. Given previous reports on platelet-synaptosomal V_max_ coupling, results further suggest an important role of neuronal 5-HT reuptake in DMN regulation.

## Introduction

The serotonin transporter (5-HTT) is abundantly expressed throughout the human body by the 5-HTT gene (*SLC6A4*). Highest amounts of 5-HTT protein can be found in the gastrointestinal tract, blood platelets, and serotonergic neurons in the brain [1]. In platelets, serotonin (5-HT) reuptake by transmembrane 5-HTTs represents the primary mechanism of 5-HT clearance from blood plasma [1]. Similarly, in serotonergic neurons, 5-HT reuptake by presynaptic 5-HTTs determines synaptic 5-HT levels [1]. The 5-HTT protein is therefore the central regulator of extracellular 5-HT levels in both platelets and serotonergic neurons [1], [2]. In the brain, 5-HTTs are involved in numerous physiologic brain functions including emotion processing [2], which is known to be altered in major depressive disorder (MDD) [3]. The clinical efficacy of selective 5-HT reuptake inhibitors (SSRIs), the first-line treatment of MDD, constitutes the leading argument for the crucial role of 5-HT neurotransmission in depression [4], [5].

Functional magnetic resonance imaging (fMRI) studies in depressed patients have contributed significantly to the biological understanding of neural changes in MDD [3], [4], [6], [7]. However, due to obvious ethical limitations alterations of neural activation have so far not been directly associated with *in vivo* functional assessments of the 5-HTT protein in humans such as maximal 5-HT uptake velocity (V_max_). Nevertheless, indirect evidence of animal or human pharmacological studies suggests that 5-HTT function is modulating neural activation in brain centers that are detectable with fMRI and have been related to MDD [7], [8], [9], [10], [11], [12], [13], [14], [15]. Firstly, pharmacological MRI (phMRI) experiments in animals have demonstrated that drug-induced 5-HT challenge leads to changes in cortical activation [8], [9]. Secondly, human phMRI studies revealed area-specific changes in BOLD signaling after SSRI administration [10], [11], [12], [13], [14], [15], [16]. Notably, short-term effects of pharmacologically increased 5-HT levels are involving a complex pattern of both BOLD signal increases and decreases in several cortical and subcortical brain regions that differ substantially from long-term effects [14], [15], [17]. Complementary, studies inducing a temporary reduction of 5-HT availability by dietary acute tryptophan depletion (ATD) have shown regionally specific effects on BOLD signaling [18], [19].

The physiologic significance of those observations remains unclear since findings of pharmacological challenge studies cannot be directly applied to human neurobiology. Hence, alternative approaches such as multimodal imaging have been taken to elucidate the relationship between neural activation and physiologic 5-HT signaling *in vivo* in healthy humans [20], [21]. While still rarely used, multimodal imaging studies have been able to relate BOLD signal changes of the amygdala and the default-mode network (DMN) to 5-HT receptor availability under physiologic conditions [20], [21]. Moreover, one study found amygdala reactivity to be predictable by amygdalar 5-HTT availability [22]. Notably, no studies investigating the relationship between 5-HTT availability within the DMN and BOLD signaling are to our knowledge available today.

Importantly, positron emission tomography (PET) imaging does not allow for functional assessments of 5-HTT transport processes such as neuronal V_max_, because it is confined to the quantification of transporter binding sites [23], [24], [25]. Hence, there is no technique available today that is capable to measure neuronal V_max_ *in vivo* in humans. Alternatively, well-established and easily assessable peripheral models of neuronal V_max_ such as platelet V_max_ can be utilized in humans [1], [24], [26], [27], [28]. The blood-brain relationship between platelet and neuronal 5-HTT is supported by several lines of evidence: (a) platelet and synaptosomal re-uptake are correlated in humans [26], [29], (b) human blood and cerebrospinal fluid (CSF) 5-HT levels exhibit similar changes, when properly assessed [30], (c) blood and brain 5-HT levels show parallel changes after administration of 5-HT-releasing drugs [31], [32], (d) platelet [33], [34], [35], [36] and neuronal [37], [38] 5-HT reuptake are heritable and genetic variation within *SLC6A4* affects 5-HT reuptake in blood cells [39], [40], [41], [42], [43], [44] as well as BOLD signaling in neural circuits [45], [46], [47], (e) depression is associated with an increased risk of cardiovascular events putatively related to platelet dysfunction [48], [49], and (f) a meta-analytical study [50] re-confirmed initial reports of an association between platelet 5-HT uptake and depression [51].

Based on above-mentioned reports on a linkage between 5-HT neurotransmission and neural activation [8], [9], [10], [11], [12], [13], [14], [15] as well as on the correlative nature between platelet and synaptosomal V_max_ [26], [29], we initiated an fMRI study in healthy subjects with the goal to investigate the putative predictive value of platelet V_max_ with respect to brain activation. Since prior studies found genetic variants within *SLC6A4* to affect 5-HTT expression [24], platelet V_max_ [41], [42], and neural activation [45], [46], [47], genetic effects have been controlled for within this study. We expected from this novel approach to gain insights how 5-HTT function is related to neural activation in brain networks of healthy subjects.

## Materials and Methods

### Subjects

Right-handed healthy native speakers of European ancestry aged between 18 and 45 years were invited to participate in this study. All subjects underwent the Structured Clinical Interview for DSM-IV Axis I disorders (SCID-I) [52] to ascertain absence of any past or present psychiatric diagnoses. Moreover, subjects had to score below eight on the 21-item version of the Hamilton Depression Scale (HAM-D) [53] to be included into the study. Additionally, a medical exam was performed comprising routine blood tests, urine drug screening, a physical medical and neurological exam as well as an electrocardiogram in order to assess the medical status of the participants. Only subjects without any current or previous psychiatric or medical illness were included in this study. All assessments were conducted at the Division of Biological Psychiatry at the Medical University of Vienna (MUV). Medical exams have been performed by physicians (C.S., G.P., and L.P.). Psychological assessments have been conducted by trained raters that were supervised by these psychiatrists. Forty-eight healthy subjects (mean age 25±4.6, 31 females) fulfilled above-mentioned inclusion criteria and were enrolled in this fMRI study. All procedures were explained to participants before obtaining written informed consent. This study has been approved by the local ethics committee in line with the Declaration of Helsinki.

### Laboratory Assessments

#### 5-HT Uptake Measurement

Blood samples (50 ml) were drawn between 9 and 10 a.m. Samples were collected by venipuncture (EDTA tubes, 1% wt/vol in saline), coded, and were transferred within a 30 min timeframe after venipuncture for analysis to the Institute of Pharmacology (Center of Biomolecular Medicine and Pharmacology, MUV). Platelet-rich plasma (PRP) was separated from blood cells by centrifugation (220 g, 15 min) and diluted with Krebs–Henseleit buffer (KH; 6.92 g NaCl, 0.35 g KCl, 0.29 g MgSO_4_.7H_2_O, 0.16 g KH_2_PO_4_, 2.1 g NaHCO_3_, 2.1 g glucose per liter, pH = 7.4). Briefly, platelet solution (40 μl) was incubated for three minutes with various 5-HT (0.03, 0.1, 0.3, 1.3, 10 μM unlabeled and [3H]5-HT [54]; specific activity 21.5–25.8 Ci/mmol; constant (0.03 μM), 500 μl KH). Nonspecific uptake was determined at 10 μM 5-HT in the presence of 1 μM paroxetine. Uptake was assessed by using a dilution technique with unlabeled 5-HT to reveal V_max_ and Km values (by recalculating and fitting the background-corrected uptake data to Michaelis- Menten kinetics, with c.p.m. values at the highest [5-HT] being 3–9 times over background). Uptake reactions were stopped by addition of 1 ml ice-cold KH and immediate centrifugation (41C, Sorvall-GLC-3, 1470 g). All experiments were done in triplicate determination.

#### Genotyping

DNA of subjects (n = 48) participating in our MRI study was isolated from EDTA blood samples using the Magna Pure LC DNA Isolation Kit (Roche). Detection of 5-HTTLPR and rs25531 were performed according to a procedure outlined elsewhere [55]. Briefly, DNA samples were subjected to polymerase chain reaction (PCR) using the primer pair 5′-TCCTCCGCTTTGGCGCCTCTTCC-3′/5′TGGGGGTTGCAGGGGAGATCCTG-3′ to amplify long/short promoter (L/S) DNA fragments. PCR products were separated on 5% Criterion Gels (Biorad) to detect long and short promoter alleles. Part of the PCR reaction was digested by HpaII (New England Biolabs) to detect rs25531. Digestion products were separated on 2% agarose gels. Genotyping resulted in the following genotype distribution: L_A_/L_A_ (n = 12), L_A_/L_G_ (n = 4), L_A_/S_A_ (n = 20), L_A_/S_G_ (n = 1), L_G_/S_A_ (n = 6), S_A_/S_A_ (n = 5). Genotypes were collapsed into a high expressing (L_A_/L_A_) (n = 12) and the remaining low expressing (S or L_G_ allele carriers) (n = 36) group for further statistical analysis [56].

### Imaging Procedures

#### Magnetic resonance imaging

MRI measurements were performed on a 3 Tesla (3T) TIM Trio scanner and a Siemens 12-channel head coil (Siemens Medical Solutions, Erlangen, Germany). Head movements were restricted using foam pillows. Functional data were acquired via a phase corrected blipped gradient echo, single shot echo planar imaging sequence (TE/TR = 42/2000 ms, 96×96 matrix, 210 mm square FOV, 20 axial slices, slice thickness = 4 mm, slice gap = 1 mm, interleaved slice acquisition).

#### Paradigm

A previously published fMRI paradigm was used [46], [57] that consists of two tasks comprising unpleasant emotional stimuli and one control task. Within the emotional tasks subjects had to match either one of two simultaneously presented fearful faces/threatening scenes with an identical target (face/scene). Scenes were selected from the International Affective Picture System (IAPS) [58], whereas faces were taken from the Ekman and Friesen data collection [59]. The control task consisted of simple geometric shapes that were matched analogously. The paradigm consisted of four blocks of scenes and four blocks of faces interleaved with nine blocks of the control task, 30 seconds each preceded by two seconds of instructions. Emotional tasks were grouped in blocks of two. Stimulus duration was five seconds. The total duration was 9.2 minutes. Stimuli were displayed using Presentation 10.3 (http://www.neurobs.com) and were projected onto a back-projection screen by a beamer placed outside the scanner room. Our contrast of interest was the weighted difference between affective pictures (faces and IAPS) versus the control condition (geometric shapes).

### Preprocessing and Statistical Analysis

#### Correlation of platelet V_max_ with local activations

Preprocessing of structural and functional MRI data was performed using standard AFNI procedures (http://afni.nimh.nih.gov/afni) [60] implemented into an R framework (http://cran-r-project.or/) [61]. Briefly, preprocessing included despiking, motion correction, alignment of the functional data to the respective anatomical dataset including template alignment (TT_N27 Talairach template), spatial filtering (Gaussian kernel, 8 mm FWHM), conversion to percent signal change, and temporal filtering (frequency band 0.009–0.1 Hz). The first five volumes were discarded to ensure that magnetization equilibrium was reached. First-level whole-brain analysis was performed within a general linear model. Movement covariates and baseline drifts were modeled as regressors of no interest. The resulting contrast estimates and their respective t-statistics were then used as input for mixed-effects multilevel analysis (MEMA) to assess correlations of BOLD activation with platelet V_max_, while controlling for age, gender, and triallelic 5-HTTLPR genotypes [62]. This approach is comparable to the conventional second level approach under conditions of normality and homogeneous effect reliability, and is superior otherwise by accounting for outliers and taking into account the reliability of effect estimates [62]. Monte Carlo simulation (10,000 iterations, dimensions: 74×87×69 grid, 2.19×2.19×2.19 mm, 9.8×9.9×9.8 mm smoothness estimated with 3dFWHMx) indicated that an initial voxel-wise threshold of p<0.005 and a minimum cluster size of 132 voxels yielded a corrected p value of 0.05. Additionally, we calculated cluster thresholds for more rigorous significance levels in order to indicate the statistical reproducibility of our results as recently recommended [63]. Required cluster sizes for multiple comparison correction at these more stringent thresholds were estimated to be at least 197 voxels for significant (p<0.005) and 245 voxels for highly significant results (p<0.001).

Confidence limits for the largest correlating clusters were estimated by bootstrap resampling, which avoids parametric assumptions about the distributions of the variables [64]. We further applied Cohen’s f^2^ in order to estimate the potential effect size of V_max_ on BOLD activity within the two most significant clusters, while controlling for age, gender, and triallelic 5-HTTLPR genotypes [65]. For these calculations, the BOLD signal was averaged across all voxels of the cluster to alleviate the effect overestimation bias that results from peak voxel selection [66].

#### Correlation of platelet V_max_ with functional connectivity measures

To assess the potential impact of platelet V_max_ on coherent functional networks, we calculated functional connectivity, which provides a robust measure to assess temporal correlations in the fMRI signal across functionally linked areas. Specifically, functional connectivity analysis explores which parts of the brain are coupled with a given “seed” region by cross-correlating the time series of the seed with all other voxels. Briefly, preprocessing of functional connectivity data consisted of despiking, motion correction, and alignment of functional data to the respective anatomical dataset including template alignment (TT_N27 Talairach template). Beyond the widely used removal of motion parameters derived from volume registration, we utilized the ANATICOR method [67] to eliminate nuisance signals estimated from eroded white matter and cerebrospinal fluid masks provided by FreeSurfer anatomical segmentation (FreeSurfer version 5.1.0 (http://surfer.nmr.mgh.harvard.edu/)) [68]. For temporal filtering, a broad frequency band (0.008–0.15 Hz) has been applied, which has previously been found to yield the highest test-retest reliability [69]. Finally, data have been blurred with a spatial Gaussian filter of 8 mm FWHM. To avoid any impact of task-related activations on connectivity estimates, correlation analysis was restricted to the control task blocks [70]. The selection of seeds was based on the whole-brain correlation analysis with platelet V_max_ including the largest positively and negatively correlated clusters as seeds for extraction of mean time series located in the medial prefrontal cortex (555 voxels, z = 3.756) and motor cortex (242 voxels, z = 6.942). Within first level analysis mean time series were regressed against the time series of the remaining voxels. The resulting single subject statistical seed to voxel correlation maps were then converted to z-scores using Fisher’s transformation formula. Single subject z-score maps were included in a second level voxel-wise regression analysis to test for possible correlation with platelet V_max_ while controlling for age, gender, and 5-HTTLPR genotype. Monte Carlo simulation (10,000 iterations, dimensions: 74×87×69 grid, 2.19×2.19×2.19 mm, 11.0×11.3×11.5 mm smoothness) indicated that an initial voxel-wise threshold of p<0.005 and a minimum cluster size of 182 voxels yielded a corrected p-value of 0.05.

To further test for the impact of platelet V_max_ on pairwise connectivity between hubs of the correlating network, seeds were defined based on our correlation analysis with platelet V_max_ in core regions of the default mode network (DMN) [71] comprising the medial prefrontal cortex (mPFC), the posterior cingulate cortex (PCC), the middle temporal gyrus as well as the temporal parietal junction. To ensure equally sized seeds, each region was defined as the 50 most significant voxels surrounding correlation peaks between V_max_ and BOLD activation. Preprocessing and second level analysis of pairwise connectivity were identical to the procedure described above. False discovery rate (FDR, q <0.05) was used for multiple comparison correction of the resulting connectivity matrix.

## Results

### Characteristics of Platelet V_max_

Mean platelet V_max_ of all investigated subjects was 0.124±0.087 pmol/10^6^ platelets/min (Table S1). Platelet V_max_ data were neither affected by age (t_(46)_ = −0.6482, p = 0.5201) nor gender (t_(36.72)_ = −0.2081, p = 0.8363). A comparison of platelet V_max_ between low (S, L_G_) and high (L_A_) expressing variants based on the triallelic classification of 5-HTTLPR and rs25531 resulted in a borderline significant (t_(15.29) = _1.8632, p = 0.0488, one-tailed) lower platelet V_max_ in S and L_G_ allele carriers compared to subjects with L_A_/L_A_ genotype in line with previous results [41], [42].

### Correlation Analysis between Local Neural Activation and Platelet V_max_

**Figure 1:**
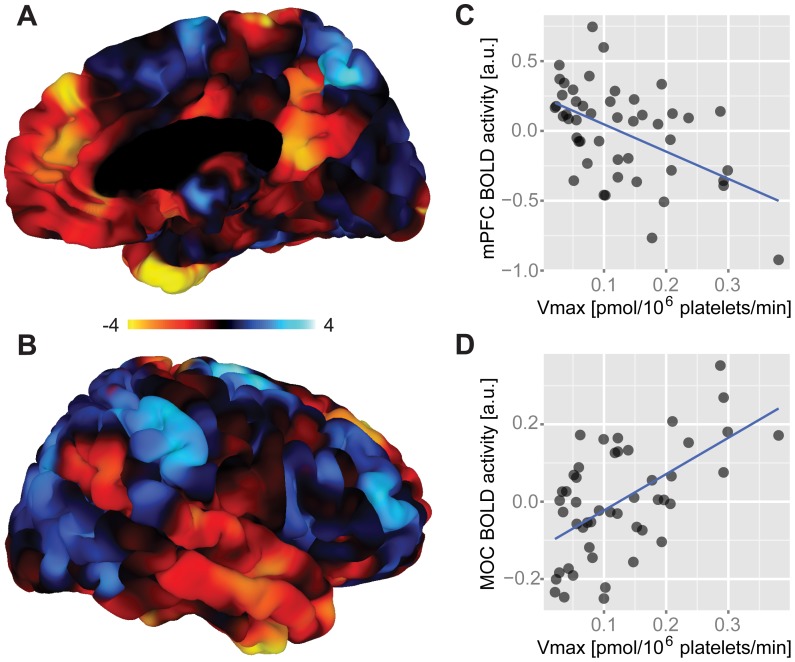
Functional brain correlates of platelet 5-HT uptake velocity. (**A–B**) Figures display right-hemispheric surface mappings of a whole-brain correlation analysis between platelet V_max_ and BOLD activity (n = 48). Significant brain areas showed positive and negative correlations. Negatively correlated clusters comprised areas of the DMN such as regions within the mPFC/ACC as well as the PCC, MTG, and ITG. Positive correlations were found in the fronto-parietal control system encompassing the CEN and SN with a significant cluster located in the right MOC and PMC. The corresponding left-hemispheric mapping is shown in Figure S2. Colorbar represents t-values. (**C**) Scatter plot shows the negative relationship between platelet V_max_ and BOLD activity averaged across the mPFC cluster (peak at [−7.7, 44.1, 27.5]). (**D**) Scatter plot shows the positive relationship between platelet V_max_ and BOLD activity averaged across the MOC cluster (peak at [20.8, −21.6, 69.1]). All analyses are controlled for age, gender and 5-HTTLPR. Serotonin, 5-HT; maximal 5-HT uptake velocity, V_max_; default mode network, DMN; medial prefrontal cortex, mPFC; anterior cingulate cortex, ACC; posterior cingulate cortex, PCC; middle temporal gyrus, MTG; inferior temporal gyrus, ITG; central executive network, CEN; salience network, SN; motor cortex, MOC; premotor cortex, PMC; blood-oxygen-level dependent, BOLD; a.u., arbitrary units.

To investigate the potential predictive value of platelet V_max_ with respect to task-related neural activation (Text S1 and Figure S1), a voxel-wise regression analysis between platelet V_max_ and whole-brain BOLD signaling controlled for age, gender, and 5-HTTLPR genotype was performed. This analysis revealed activation clusters that significantly correlated negatively as well as positively with platelet V_max_. Negatively correlated clusters comprised areas within the mPFC such as the anterior cingulate cortex (ACC) as well as the posterior cingulate cortex, precuneus, and left middle and inferior temporal gyrus (Figure 1, Figure S2, Figure S3, and Table 1). Importantly, all of these regions are neural nodes of the well-characterized DMN, which is suppressed during task performance and active during rest [71], [72], [73]. A positively correlated cluster encompassed the right motor and premotor cortex (Figure 1, Figure S2, Figure S3, and Table 1).

**Table 1:** Correlation analysis between maximal platelet 5-HT uptake velocity (V_max_) and neuronal activation (n = 48).

Region	Hemisphere	Size	z	p	x	y	z
mPFC, ACC	R/L	555	−3.756	<0.001***	−7.7	44.1	27.5
MOC, PMC	R	242	6.942	<0.001**	20.8	−21.6	69.1
PCC, PRE	R/L	189	−3.503	<0.001*	3.3	−47.8	14.4
MTG, ITG	L	167	−4.542	<0.001*	−55.8	−10.6	−9.7

Medial prefrontal cortex, mPFC; anterior cingulate cortex, ACC; motor cortex, MOC; premotor cortex, PMC; posterior cingulate cortex, PCC; precuneus, PRE; middle temporal gyrus, MTG; inferior temporal gyrus, ITG; p, uncorrected p value; significance level after correction for multiple comparisons based on recent recommendations [63]: ***p<0.001; **p<0.005; *p<0.05; x, y, z are coordinates in Talairach space; L, left hemisphere; R, right hemisphere; cluster size expressed as number of voxels.

Bootstrap estimation of the two peak clusters exhibiting opposite correlations revealed a Pearson correlation coefficient of −0.55 (bias = 0.01; std. error = 0.12; 95% confidence interval: −0.78, −0.34) for the medial prefrontal cortex cluster (Figure S4) and 0.63 (bias = 0.01; std. error = 0.09; 95% confidence interval: 0.45, 0.80) for the motor cortex cluster (Figure S5).

With respect to effect sizes of these findings, Cohen’s f^2^ indicated large effects for the medial prefrontal cortex (Cohen’s f^2^ = 0.40) and the motor cortex (Cohen’s f^2^ = 0.58). However, it should be noted that effect size calculations are prone to an overestimation in fMRI studies as recently pointed out [66].

### Functional Connectivity Analysis

**Figure 2:**
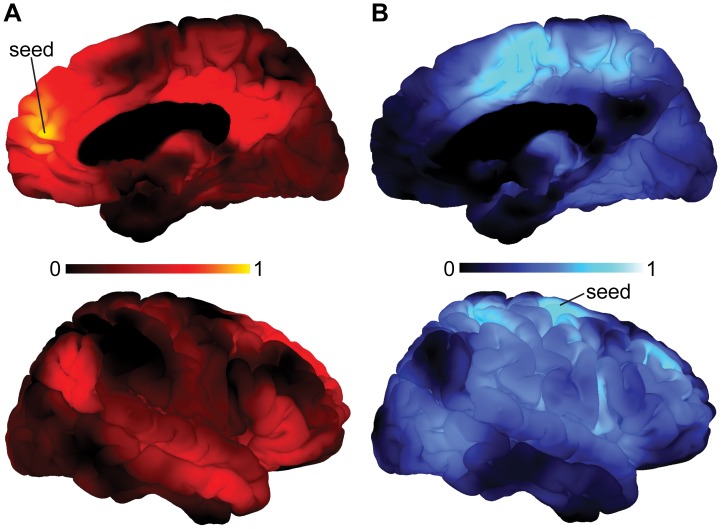
Functional connectivity analysis of the mPFC and MOC cluster. (**A**) Figures display right-hemispheric surface mappings of a functional connectivity analysis utilizing the mPFC cluster as seed region. Areas showing increased coupling with the mPFC comprised the PCC, precuneus, MTG and temporal parietal junction. All of these areas correspond to core regions of the DMN. (**B**) Figures display right-hemispheric surface mappings of a functional connectivity analysis utilizing the MOC cluster as seed region. Areas showing increased coupling with the MOC have been found exclusively in brain regions that spatially correspond to the CEN or SN, while functional coupling with the DMN was absent. The corresponding left-hemispheric mappings are shown in Figure S6. Colorbars represent mean Pearson’s r. Medial prefrontal cortex, mPFC; posterior cingulate cortex, PCC; middle temporal gyrus, MTG; motor cortex, MOC; default-mode network, DMN; central executive network, CEN; salience network, SN.

To further investigate network characteristics of above reported regions showing significant correlations with platelet V_max_, two *post hoc* functional connectivity analyses were conducted. The first analysis utilized the cluster showing maximal negative correlation with platelet V_max_ as seed region, whereas for the second analysis the maximally positively correlated cluster was chosen. Interestingly, whole-brain functional connectivity analyses for both seeds revealed two large and spatially highly complementary neural systems (Figure 2, Figure S6, Table S2, and Table S3). Briefly, areas showing increased coupling with the mPFC comprised the posterior cingulate cortex, precuneus, middle temporal gyrus, and temporal parietal junction, all of which correspond to core regions of the DMN that were negatively correlated to platelet V_max_ in this study [71], [72]. In contrast, areas showing increased coupling with the motor cortex, which was positively correlated to platelet V_max,_ have been found exclusively in brain regions that spatially correspond to the central executive (CEN) or salience (SN) network [74], [75], [76], [77], while functional coupling with the DMN was absent.

Notably, there was no impact of platelet V_max_ on these functional connectivity measures. Additionally, we tested for the potential impact of platelet V_max_ on pairwise intrinsic connectivity of the DMN, which was not supported by our data (Figure S7). Summarizing, our results highlight that increases in platelet V_max_ predict global DMN suppression in healthy humans.

## Discussion

The present study provides evidence that platelet V_max_ predicts global DMN activation in healthy subjects. Given previous reports on platelet-synaptosomal V_max_ coupling, results of this study further suggest an important role of neuronal 5-HT reuptake in DMN regulation.

A significant negative correlation between platelet V_max_ and neural activation has predominantly been found within 5-HTT-rich cortical areas in the midline of the brain [23], [78] (Figure 1, Figure S2, Figure S3, Figure S8, and Table 1). Brain regions showing an inverse relationship between platelet V_max_ and neural activation comprised areas of the mPFC including the ACC as well as the PCC, precuneus, and middle as well as inferior temporal gyrus (Figure 1, Figure S2, Figure S3), all of which are known to be core regions of the DMN [71], [72], [73]. Notably, the largest cluster (mPFC) even survived the application of a more rigorous significance threshold (p<0.001, corrected for multiple comparisons), which indicates high statistical reproducibility [63].

Briefly, the DMN consists of above-mentioned brain regions that show marked activity during rest and low activation during focused attention on the external environment [71], [72], [73], [79]. Prevailing hypotheses conceptualize the physiological role of the DMN as primarily related to self-referential thought (e.g. internal mentation, day dreaming, etc.) [79]. A major feature of the DMN is its suppression during attention-demanding tasks. This anti-correlative suppression is necessary for proper task performance and avoids ‘mind wandering’ resulting from conflicting self-referential computations [79]. Lacking DMN suppression during attention-demanding tasks is of clinical importance and has been linked to increased rumination in depressed patients [80], [81].

The observed coupling between platelet V_max_ and core regions of the DMN is of specific interest, because a thorough understanding of the neurochemical regulation of the DMN is still missing [20]. Available evidence indicates that monoamines such as dopamine and 5-HT play a major regulatory role [16], [20], [82], [83], [84], [85]. Dopamine affects DMN coupling with attention networks [82] and the application of dopaminergic agents such as modafinil leads to DMN suppression [83]. An analogous suppression of the DMN can be achieved by serotonergic agents such as the 5-HT_2A_/5-HT_2C_ agonist psilocybin [84]. Moreover, 5-HT_1A_ autoreceptor binding has been shown to be anti-correlated with PCC activation, while opposing effects were found for retrosplenial and medial prefrontal DMN regions [20]. A similar regionally-specific effect of serotonergic neurotransmission on DMN activity has been reported in a tryptophan depletion study [85]. Given the spatial co-occurrence between cortical regions of high 5-HTT densities and nodes of the DMN [23], [78] (Text S1 and Figure S8), it is interesting that 5-HT reuptake inhibition with escitalopram or combined 5-HT and norepinephrine inhibition with duloxetine have been demonstrated to decrease DMN coupling in human phMRI studies [16], [86].

Notably, pharmacological challenge studies [16], [86] are not well suited to study the physiological effects of 5-HT reuptake on DMN activation. However, the absence of any effect would have made the existence of a physiologic coupling between 5-HT reuptake and DMN activity implausible. Hence, the studies of *Van der Ven et al*. [16] and *Van Wingen et al.* [86] together with other studies [20], [84], [85] clearly indicate an important role of brain 5-HT in the regulation of the DMN. Our finding of a negative correlation between platelet V_max_ and DMN activity provides further evidence for a possible impact of 5-HT transport processes in the regulation of the DMN. Moreover, the observed coupling between platelet V_max_ and neural activity indirectly supports the idea that platelet 5-HT reuptake can indeed be utilized as surrogate marker of neural 5-HT reuptake, which is in accordance with studies showing a coupling between platelet and synaptosomal 5-HT reuptake [26] as well as others that demonstrated 5-HTT-mediated effects on BOLD signaling [8], [9], [10], [11], [12], [13], [14], [15].

While the majority of brain regions showed a negative correlation with platelet V_max_ in our study, we report a single cluster of neural activation that correlates positively with platelet V_max_ (Figure 1, Figure S3, Figure S5, and Table 1)_._ This cluster comprises the primary motor and premotor cortex that is related to motor function or motor learning [87]. It is noteworthy that these regions are engaged during the performance of externally-oriented tasks and are therefore active, when the DMN is typically suppressed [75]. This is in line with our functional connectivity analysis of this cluster that revealed no connectivity to the DMN, but a widely distributed network of significantly coupled cortical regions belonging to the CEN and SN (Figure 2 and Figure S6). Both CEN and SN are large brain networks of the fronto-parietal control system that have intensively been investigated using resting-state and task-based fMRI [74], [75], [76], [77]. While the CEN has been related to high-level cognitive functions such as working memory and attention control, the SN responds to behaviorally salient events and has been implicated in switching between endogenously and exogenously driven mental activity [77]. Anatomically, dorsolateral prefrontal and posterior parietal cortices have been identified as nodes of the CEN, whereas the SN is anchored in the anterior insular and dorsal anterior cingulate cortex [77].

This study further investigated the possibility that variability in platelet V_max_ might explain changes of DMN intrinsic connectivity. This question was asked, because recent studies reported a modulation of medial DMN intrinsic connectivity after pharmacological challenge with serotonergic drugs [16], [84], [86], [88]. In contrast, our results do not support the idea that synaptic 5-HT reuptake is altering the pair-wise coupling of DMN nodes (Figure S7). Given the inconsistency of reported changes of connections in between DMN nodes [16], [84], [86], [89], we argue that observed alterations are likely rather reflecting effects related to the specific drug being used than effects related to synaptic 5-HT reuptake in general. Moreover, the reported findings might only apply to more extreme changes of synaptic 5-HT reuptake that go along with pharmacological interventions. Hence, we are tempted to speculate that 5-HT reuptake is not affecting intrinsic DMN connectivity *in vivo* under physiologic conditions.

Previous studies reported effects of genetic variability within the 5-HTT gene (*SLC6A4*) on 5-HTT expression [90], platelet V_max_ [41], [42], and BOLD signaling [45], [46]. Since both platelet and neural 5-HTTs are encoded by the same gene, we investigated the potential impact of genetic variants within *SLC6A4* on platelet V_max_ and the observed correlation between platelet V_max_ and BOLD signaling. The comparison of platelet V_max_ between low (S, L_G_) and high (L_A_) expressing variants of 5-HTTLPR and rs25531 revealed lower platelet V_max_ in S and L_G_ allele carriers in comparison to subjects with L_A_/L_A_ genotype. This finding reflects the lower transcription efficacy of the S allele [56] thereby resembling previous reports [41], [42]. Importantly, we found significant correlations between V_max_ and the BOLD signal in DMN areas despite controlling for triallelic 5-HTTLPR, what suggests that this finding cannot be attributed to 5-HTTLPR or rs25531 alone. It is noteworthy, however, that stress-dependent promoter methylation of 5-HTTLPR alleles might significantly vary between platelet precursor cells and neural cells thereby underscoring epigenetic differences in blood and brain [91], [92], [93], [94]. Hence, our finding points towards the possible importance of other biological factors affecting both, platelet and neuronal V_max_. Possible candidates among others would be genetic variability within *SEC24* that has been demonstrated to affect the correct delivery of 5-HTTs to the cell surface [95] or cytokine signaling that impacts on 5-HTT availability in platelets and neurons [96], [97].

Some precautions should be considered within the context of this study. Firstly, we assessed platelet V_max_, which was used as surrogate marker of synaptosomal V_max_ in this study, due to the fact that synaptosomal V_max_ cannot be measured *in vivo* in humans [1], [24], [26], [27]. The rationale for using platelet V_max_ as estimator of synaptosomal V_max_ is further based on studies that reported a coupling between platelet and synaptosomal V_max_ [26], [29] as well as blood and CSF 5-HT levels with [31], [32] or without [30] 5-HT challenge. Moreover, platelet and synaptosomal V_max_ are heritable [33], [34], [35], [36] and genetic variation within *SLC6A4* affects 5-HT reuptake in platelets [39], [40],[41],[42],[43],[44] as well as neural signaling [45], [46]. Secondly, conclusions drawn within this investigation are derived from findings of phMRI studies that reported tight associations between 5-HT neurotransmission and BOLD signaling in humans [10], [11], [12], [13], [14], [15], [16] and animals [8], [9]. Since both *a priori* assumptions (platelet and synaptosomal V_max_ coupling as well as measurable effects of 5-HT reuptake on BOLD signaling) are founded on multiple scientific reports conducted in the past, we are confident that our conclusions are not too far-fetched. However, replication studies are needed before final conclusions with respect to the reported association between platelet V_max_ and DMN activation can be drawn. Another interesting question is why the subgenual cingulate cortex (sACC) [98], [99], which shows the highest cortical 5-HTT binding in *post mortem* studies [78] and in own PET data (Figure S9, Text S1, and Table S4), has not been found to be the main peak of correlation with platelet V_max_ within this study given its repeated implication in depression neurobiology [98] and therapy [99]. Aside from the fact that the sACC was actually part of the medial prefrontal cluster that correlated significantly with platelet V_max,_ it is noteworthy to highlight that this region is located in a part of the brain that is prone to high data variance due to susceptibility artifacts. This has likely led to a disadvantageous signal-to-noise ratio in this region and a higher chance to statistically fail in a whole-brain analysis. Hence, replication studies with larger sample size are clearly needed to re-evaluate the specific role of the sACC in this context.

Summarizing, we found that platelet V_max_ is predicting global DMN activity in healthy humans *in vivo*. Considering previous studies that have demonstrated a tight coupling between platelet and synaptosomal V_max_, this study suggests an important role of neuronal 5-HT reuptake in DMN regulation. The feasibility to use a blood measure to predict a well-characterized neural pattern, as shown in this study, encourages ongoing studies investigating putative blood biomarkers of neural phenotypes.

## Supporting Information

Figure S1 **Task activation.** Brain activation during processing of emotional pictures (faces+IAPS), contrasted against processing of neutral pictures (geometric shapes). Data is displayed at p<0.005. Colorbar represents z-scores.
(PDF)

Figure S2 **Functional brain correlates of platelet 5-HT uptake velocity.** Figures display left-hemispheric surface mappings of a whole-brain correlation analysis between platelet V_max_ and BOLD activity (n = 48). Significant brain areas showed positive and negative correlations. Negatively correlated clusters comprised areas of the DMN such as regions within the mPFC/ACC as well as the PCC, MTG, and ITG. Positive correlations were found in the fronto-parietal control system encompassing the CEN and SN. The corresponding right-hemispheric mapping is shown in Figure 1. Colorbar represents t-values. All analyses are controlled for age, gender and 5-HTTLPR. Serotonin, 5-HT; maximal 5-HT uptake velocity, V_max_; medial prefrontal cortex, mPFC; anterior cingulate cortex, ACC; posterior cingulate cortex, PCC; middle temporal gyrus, MTG; inferior temporal gyrus, ITG; motor cortex, MOC; premotor cortex, PMC; default-mode network, DMN; central executive network, CEN; salience network, SN.
(PDF)

Figure S3 **Functional brain correlates of platelet 5-HT uptake velocity.** Figures display a whole-brain correlation analysis between platelet V_max_ and BOLD activity (n = 48) corrected for age, gender, and 5-HTTLPR genotype (threshold p<0.005, colorbar represents t-values). Significant brain areas showed positive and negative correlations. Negatively correlated clusters comprised areas of the DMN such as regions within the mPFC including the ACC as well as the PCC, MTG, and ITG. Positive correlations were found in the fronto-parietal control system encompassing the CEN and SN with a significant cluster located in the right MOC and PMC. The corresponding surface mappings are shown in Figure 1 and Figure S2. Serotonin, 5-HT; maximal 5-HT uptake velocity, Vmax; medial prefrontal cortex, mPFC; anterior cingulate cortex, ACC; posterior cingulate cortex, PCC; middle temporal gyrus, MTG; inferior temporal gyrus, ITG; motor cortex, MOC; premotor cortex, PMC; default-mode network, DMN; central executive network, CEN; salience network, SN.
(PDF)

Figure S4 **Bootstrap distribution of the correlation between the BOLD signal in the medial prefrontal cortex (mPFC) cluster and platelet serotonin uptake velocity (V_max_).** The original statistic is indicated in red.
(PDF)

Figure S5 **Bootstrap distribution of the correlation between the BOLD signal in the motor cortex (MOC) cluster and platelet serotonin uptake velocity (V_max_).** The original statistic is indicated in red.
(PDF)

Figure S6 **Functional connectivity analysis of the mPFC and MOC cluster.** **(A)** Figures display left-hemispheric surface mappings of a functional connectivity analysis utilizing the mPFC cluster as seed region. Areas showing increased coupling with the mPFC comprised the PCC, precuneus, MTG and temporal parietal junction. All of these areas correspond to core regions of the DMN. **(B)** Figures display left-hemispheric surface mappings of a functional connectivity analysis utilizing the MOC cluster as seed region. Since this seed is located in the right hemisphere, it is not depicted in the figure. Areas showing increased coupling with the MOC have been found exclusively in brain regions that spatially correspond to the CEN or SN, while functional coupling with the DMN was absent. The corresponding right-hemispheric mappings are shown in Figure 2. Colorbars represent mean Pearson’s r. Medial prefrontal cortex, mPFC; posterior cingulate cortex, PCC; middle temporal gyrus, MTG; motor cortex, MOC; default-mode network, DMN; central executive network, CEN; salience network, SN.
(PDF)

Figure S7 **Correlation of platelet V_max_ and intrinsic connectivity of the DMN.** The connectivity matrix displays z-scores of the correlation between platelet serotonin uptake velocity (V_max_) and the intrinsic connectivity of the default mode network (DMN). There was no significant correlation between platelet V_max_ and intrinsic connectivity of the DMN. False discovery rate (FDR) was used for multiple comparison correction (q <0.5). Medial prefrontal cortex, mPFC; posterior cingulate cortex, PCC; right middle temporal gyrus, r MTG; left middle temporal gyrus, l MTG; left temporal parietal junction, l TPJ; right temporal parietal junction, r TPJ.
(PDF)

Figure S8 **Cortical expression map of *SLC6A4* of all available adults of European ancestry within the Allen Human Brain Atlas collection.** Illustrated are ranked and averaged z-scores for cortical regions. Zero on the Y-axis refers to average cortical expression in the brain. Please note that all DMN regions are exhibiting increased expression values.
(PDF)

Figure S9 **Surface mapping displays regional areas of increased serotonin transporter (5-HTT) availability within the cingulate cortex (CC) in healthy subjects (n = 8).** [11C]DASB PET data have been tested against deviation from mean CC binding. It is noteworthy that the subgenual anterior cingulate cortex (sACC) contains the largest cluster of significant voxels within the whole CC. Colorbar represents t-values.
(PDF)

Table S1 **Differences in maximal platelet serotonin (5-HT) uptake velocity (V_max_, in pmol/10^6^ platelets/min) and age between male and female subjects.** n – number of subjects. std – standard deviation.
(DOC)

Table S2 **Regions exhibiting maximal connectivity to the medial prefrontal cortex cluster, thresholded at Pearson’s r >0.5.** ^a^Pearson’s r. ^b^Coordinates are given in Talairach space.
(DOC)

Table S3 **Regions exhibiting maximal connectivity to the motor cortex cluster, thresholded at Pearson’s r >0.5. ^a^Pearson’s r. ^b^Coordinates are given in Talairach space.**
(DOC)

Table S4 **Clusters of locally significantly increased serotonin transporter (5-HTT) availability compared to the whole cingulate cortex (CC).** ^a^Uncorrected z-scores next to corresponding FWE-corrected one-tailed p values. ^b^Coordinates are given in Talairach space.
(DOC)

Text S1 **Supplementary methods.**
(DOC)
