# Supplementary figures and images for: Platelet Serotonin Transporter Function Predicts Default-Mode Network Activity
Source: PLoS One. 2014 Mar 25;9(3):e92543. doi: 10.1371/journal.pone.0092543 (PMC3965432; doi:10.1371/journal.pone.0092543)

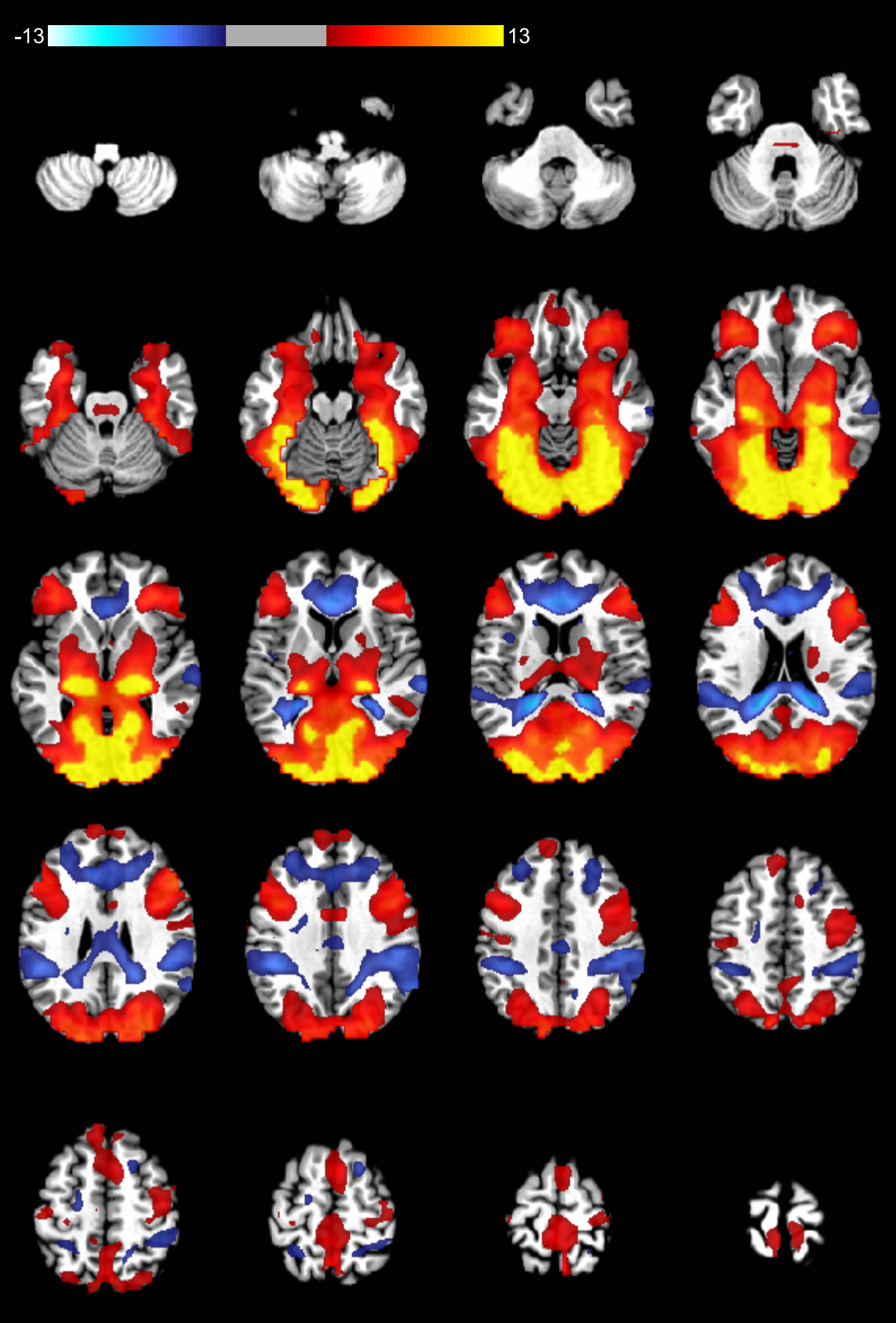

Supplement: Figure S1 — Task activation. Brain activation during processing of emotional pictures (faces+IAPS), contrasted against processing of neutral pictures (geometric shapes). Data is displayed at p<0.005. Colorbar represents z-scores. (PDF) [file pone.0092543.s001.pdf]

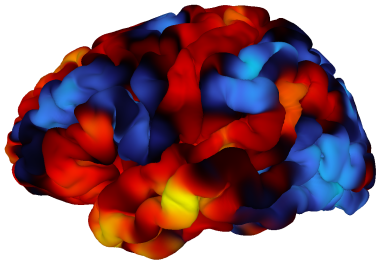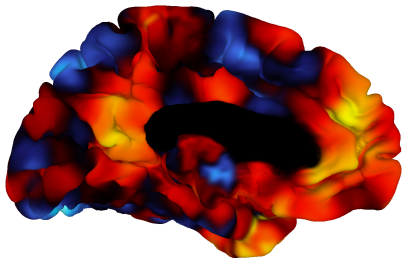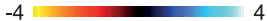

Supplement: Figure S2 — Functional brain correlates of platelet 5-HT uptake velocity. Figures display left-hemispheric surface mappings of a whole-brain correlation analysis between platelet Vmax and BOLD activity (n = 48). Significant brain areas showed positive and negative correlations. Negatively correlated clusters comprised areas of the DMN such as regions within the mPFC/ACC as well as the PCC, MTG, and ITG. Positive correlations were found in the fronto-parietal control system encompassing the CEN and SN. The corresponding right-hemispheric mapping is shown in Figure 1. Colorbar represents t-values. All analyses are controlled for age, gender and 5-HTTLPR. Serotonin, 5-HT; maximal 5-HT uptake velocity, Vmax; medial prefrontal cortex, mPFC; anterior cingulate cortex, ACC; posterior cingulate cortex, PCC; middle temporal gyrus, MTG; inferior temporal gyrus, ITG; motor cortex, MOC; premotor cortex, PMC; default-mode network, DMN; central executive network, CEN; salience network, SN. (PDF) [file pone.0092543.s002.pdf]

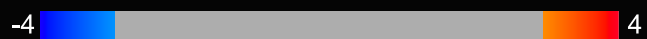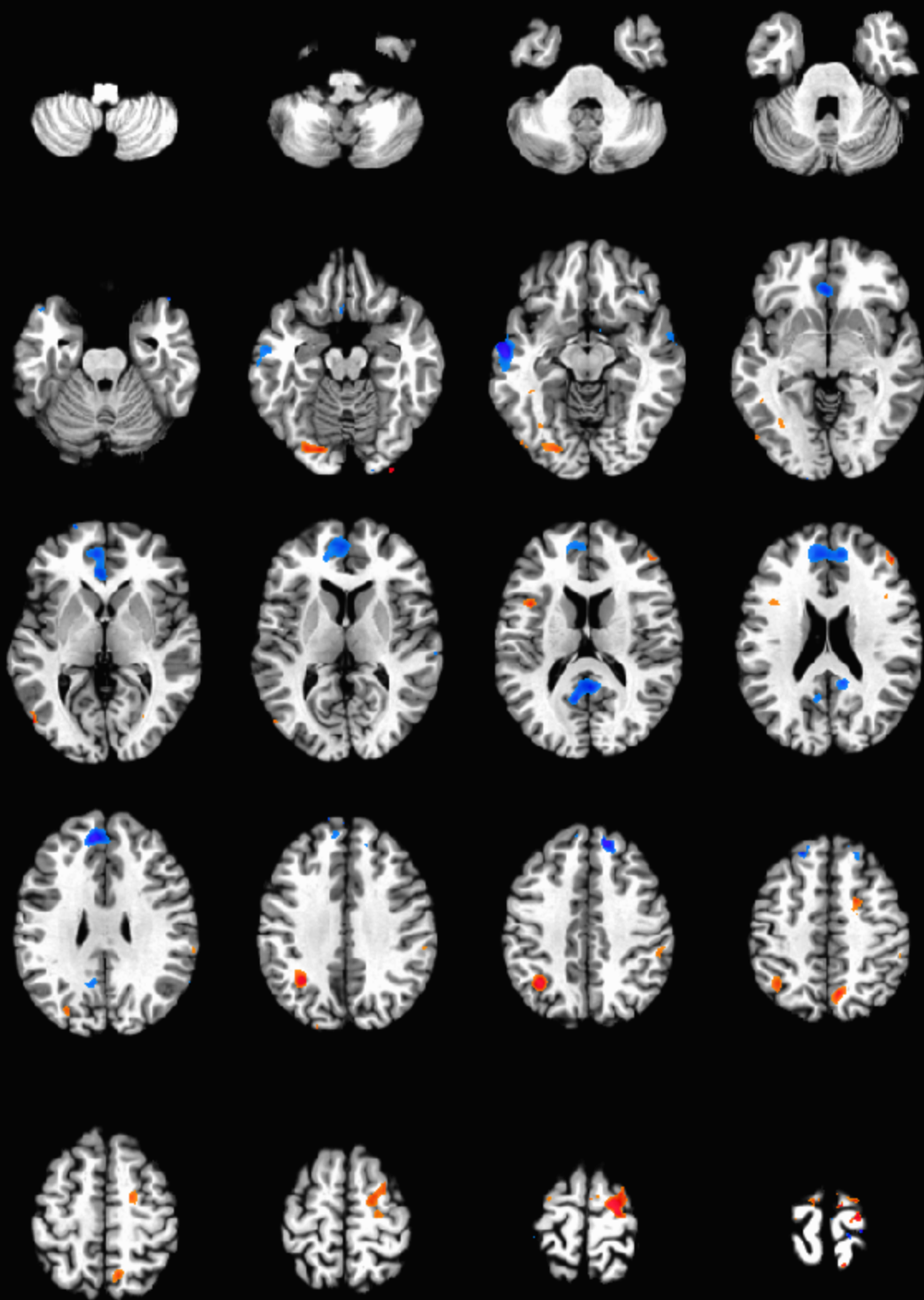

Supplement: Figure S3 — Functional brain correlates of platelet 5-HT uptake velocity. Figures display a whole-brain correlation analysis between platelet Vmax and BOLD activity (n = 48) corrected for age, gender, and 5-HTTLPR genotype (threshold p<0.005, colorbar represents t-values). Significant brain areas showed positive and negative correlations. Negatively correlated clusters comprised areas of the DMN such as regions within the mPFC including the ACC as well as the PCC, MTG, and ITG. Positive correlations were found in the fronto-parietal control system encompassing the CEN and SN with a significant cluster located in the right MOC and PMC. The corresponding surface mappings are shown in Figure 1 and Figure S2. Serotonin, 5-HT; maximal 5-HT uptake velocity, Vmax; medial prefrontal cortex, mPFC; anterior cingulate cortex, ACC; posterior cingulate cortex, PCC; middle temporal gyrus, MTG; inferior temporal gyrus, ITG; motor cortex, MOC; premotor cortex, PMC; default-mode network, DMN; central executive network, CEN; salience network, SN. (PDF) [file pone.0092543.s003.pdf]

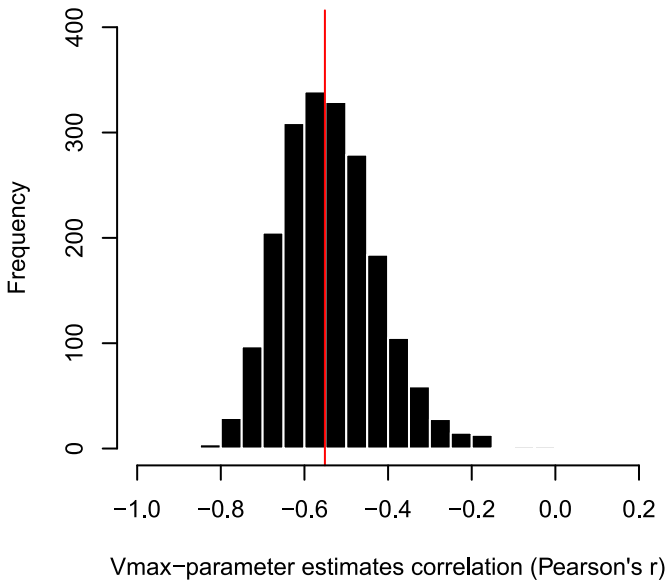

Supplement: Figure S4 — Bootstrap distribution of the correlation between the BOLD signal in the medial prefrontal cortex (mPFC) cluster and platelet serotonin uptake velocity (Vmax). The original statistic is indicated in red. (PDF) [file pone.0092543.s004.pdf]

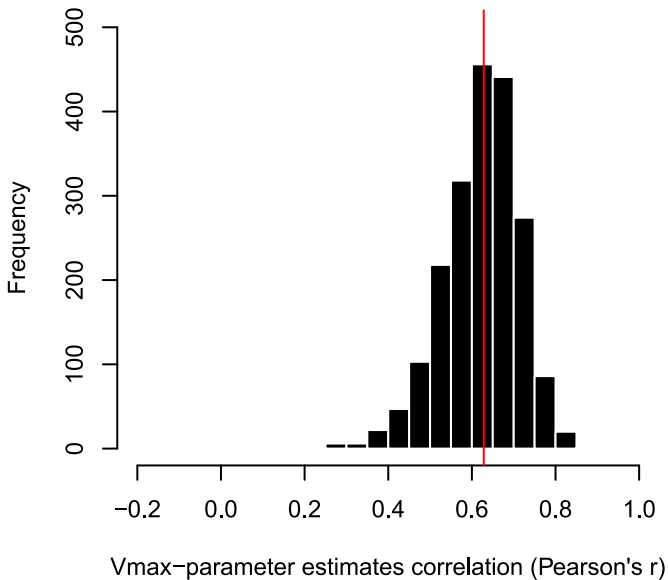

Supplement: Figure S5 — Bootstrap distribution of the correlation between the BOLD signal in the motor cortex (MOC) cluster and platelet serotonin uptake velocity (Vmax). The original statistic is indicated in red. (PDF) [file pone.0092543.s005.pdf]

**A**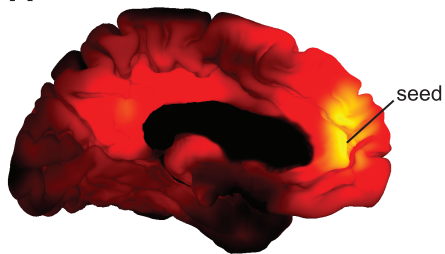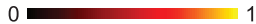**B**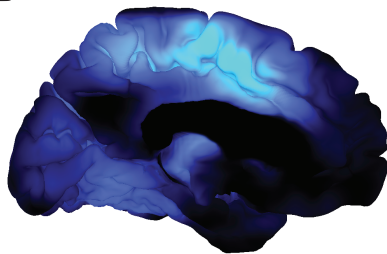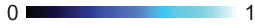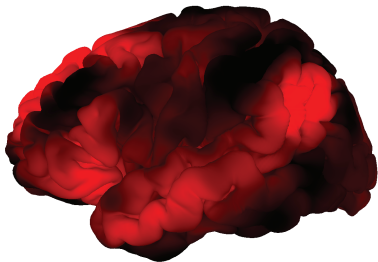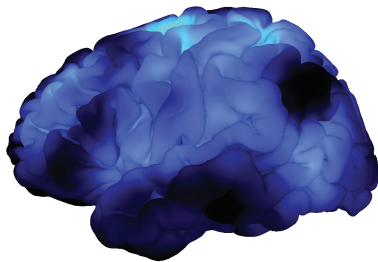

Supplement: Figure S6 — Functional connectivity analysis of the mPFC and MOC cluster. (A) Figures display left-hemispheric surface mappings of a functional connectivity analysis utilizing the mPFC cluster as seed region. Areas showing increased coupling with the mPFC comprised the PCC, precuneus, MTG and temporal parietal junction. All of these areas correspond to core regions of the DMN. (B) Figures display left-hemispheric surface mappings of a functional connectivity analysis utilizing the MOC cluster as seed region. Since this seed is located in the right hemisphere, it is not depicted in the figure. Areas showing increased coupling with the MOC have been found exclusively in brain regions that spatially correspond to the CEN or SN, while functional coupling with the DMN was absent. The corresponding right-hemispheric mappings are shown in Figure 2. Colorbars represent mean Pearson’s r. Medial prefrontal cortex, mPFC; posterior cingulate cortex, PCC; middle temporal gyrus, MTG; motor cortex, MOC; default-mode network, DMN; central executive network, CEN; salience network, SN. (PDF) [file pone.0092543.s006.pdf]

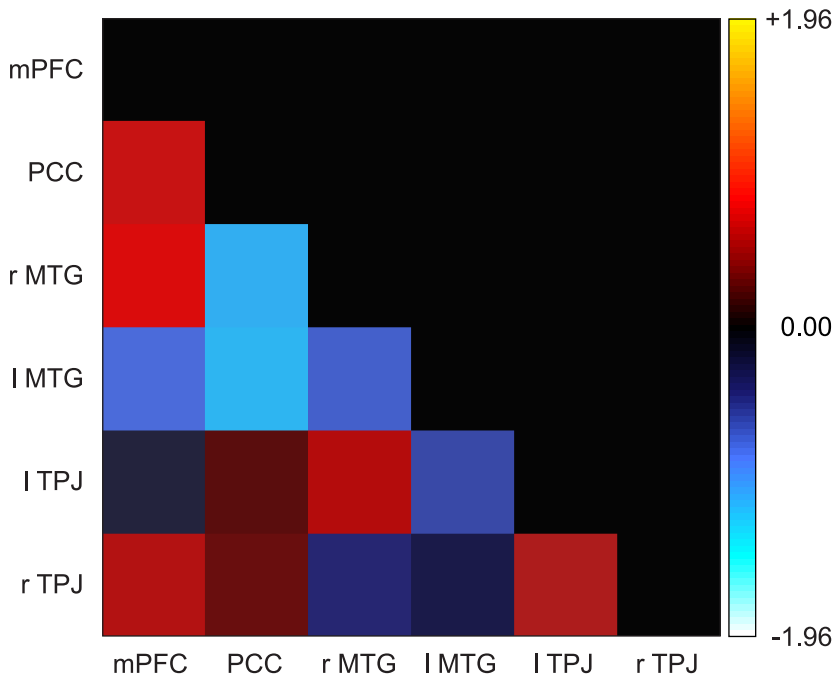

Supplement: Figure S7 — Correlation of platelet Vmax and intrinsic connectivity of the DMN. The connectivity matrix displays z-scores of the correlation between platelet serotonin uptake velocity (Vmax) and the intrinsic connectivity of the default mode network (DMN). There was no significant correlation between platelet Vmax and intrinsic connectivity of the DMN. False discovery rate (FDR) was used for multiple comparison correction (q <0.5). Medial prefrontal cortex, mPFC; posterior cingulate cortex, PCC; right middle temporal gyrus, r MTG; left middle temporal gyrus, l MTG; left temporal parietal junction, l TPJ; right temporal parietal junction, r TPJ. (PDF) [file pone.0092543.s007.pdf]

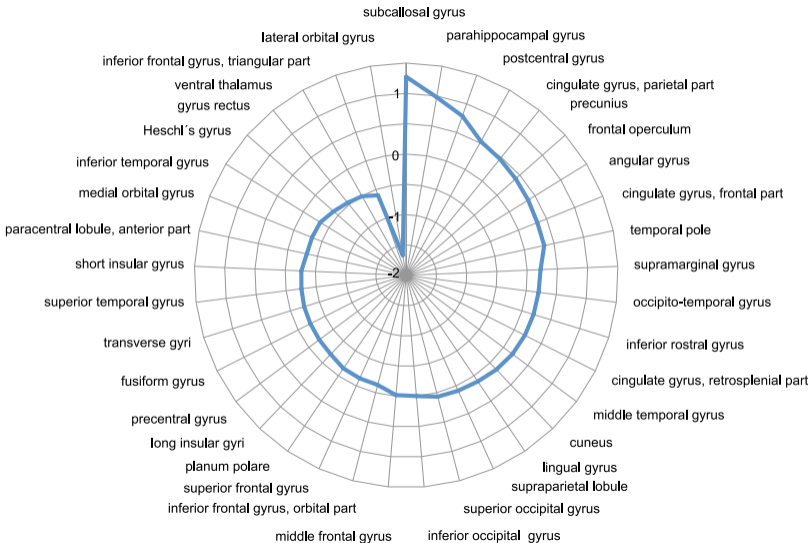

Supplement: Figure S8 — Cortical expression map of SLC6A4 of all available adults of European ancestry within the Allen Human Brain Atlas collection. Illustrated are ranked and averaged z-scores for cortical regions. Zero on the Y-axis refers to average cortical expression in the brain. Please note that all DMN regions are exhibiting increased expression values. (PDF) [file pone.0092543.s008.pdf]

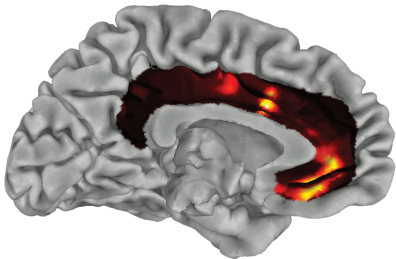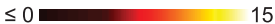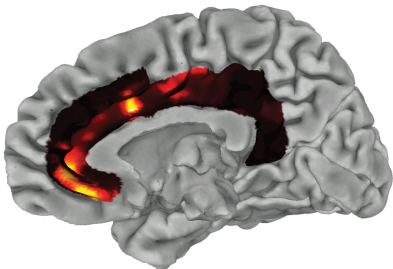

Supplement: Figure S9 — Surface mapping displays regional areas of increased serotonin transporter (5-HTT) availability within the cingulate cortex (CC) in healthy subjects (n = 8). [11C]DASB PET data have been tested against deviation from mean CC binding. It is noteworthy that the subgenual anterior cingulate cortex (sACC) contains the largest cluster of significant voxels within the whole CC. Colorbar represents t-values. (PDF) [file pone.0092543.s009.pdf]
