# Supplementary material for: Platelet Serotonin Transporter Function Predicts Default-Mode Network Activity
Source: PLoS One. 2014 Mar 25;9(3):e92543. doi: 10.1371/journal.pone.0092543 (PMC3965432; doi:10.1371/journal.pone.0092543)
Supplement: Table S1 — Differences in maximal platelet serotonin (5-HT) uptake velocity (Vmax, in pmol/106 platelets/min) and age between male and female subjects. n – number of subjects. std – standard deviation. (DOC) [file pone.0092543.s010.doc]

|  | All |  |  |  |  | Female | |  | Male |  |  |
| --- | --- | --- | --- | --- | --- | --- | --- | --- | --- | --- | --- |
|  | n | mean | std | t | p | n | mean | std | n | mean | std |
| V_max_ | 48 | 0.12 | 0.09 | -0.21 | 0.84 | 31 | 0.12 | 0.10 | 17 | 0.12 | 0.08 |
| Age | 48 | 24.5 | 3.70 | -0.65 | 0.52 | 31 | 24.68 | 3.36 | 17 | 24.25 | 4.22 |

**Table S1.** Differences in maximal platelet serotonin (5-HT) uptake velocity (V_max_, in pmol/10^6^ platelets/min) and age between male and female subjects. n – number of subjects. std – standard deviation
