# Supplementary material for: Platelet Serotonin Transporter Function Predicts Default-Mode Network Activity
Source: PLoS One. 2014 Mar 25;9(3):e92543. doi: 10.1371/journal.pone.0092543 (PMC3965432; doi:10.1371/journal.pone.0092543)
Supplement: Table S2 — Regions exhibiting maximal connectivity to the medial prefrontal cortex cluster, thresholded at Pearson’s r >0.5. aPearson’s r. bCoordinates are given in Talairach space. (DOC) [file pone.0092543.s011.doc]

| Region | Hemisphere | r^a^ | x^b^ | y | z |
| --- | --- | --- | --- | --- | --- |
| Medial prefrontal cortex | L/R | 0.939 | -3.3 | 48.4 | 12.2 |
| Posterior cingulate cortex | L/R | 0.758 | -5.5 | -47.8 | 27.5 |
| Temporal parietal junction | L | 0.631 | -44.8 | -56.6 | 25.3 |
| Inferior frontal gyrus | L | 0.569 | -38.3 | 28.8 | -3.1 |
| Temporal parietal junction | R | 0.581 | 47 | -56.6 | 23.1 |
| Middle temporal gyrus | L | 0.503 | -55.8 | -15 | -7.5 |
| Inferior frontal gyrus | R | 0.534 | 29.5 | 17.8 | -7.5 |
| Middle temporal gyrus | R | 0.511 | 53.6 | 2.5 | -18.4 |
|  |  |  |  |  |  |
|  |  |  |  |  |  |

**Table S2.** Regions exhibiting maximal connectivity to the medial prefrontal cortex cluster, thresholded at Pearson´s r > 0.5. ^a^ Pearson´s r. ^b^ Coordinates are given in Talairach space
