# Supplementary material for: Platelet Serotonin Transporter Function Predicts Default-Mode Network Activity
Source: PLoS One. 2014 Mar 25;9(3):e92543. doi: 10.1371/journal.pone.0092543 (PMC3965432; doi:10.1371/journal.pone.0092543)
Supplement: Table S3 — Regions exhibiting maximal connectivity to the motor cortex cluster, thresholded at Pearson’s r >0.5. aPearson’s r. bCoordinates are given in Talairach space. (DOC) [file pone.0092543.s012.doc]

| Region | Hemisphere | r^a^ | x^b^ | y | z |
| --- | --- | --- | --- | --- | --- |
| Motor and premotor cortex | R | 0.934 | 20.8 | -10.6 | 60.3 |
| Medial superior frontal cortex | R | 0.646 | 31.7 | 35.3 | 34.1 |
| Medial superior frontal cortex | L | 0.608 | -31.7 | 33.1 | 34.1 |
| Superior temporal gyrus | L | 0.517 | -47 | -65.3 | 10 |
| Thalamus | L | 0.531 | -7.7 | -17.2 | 12.2 |
| Thalamus | R | 0.518 | 7.7 | -17.2 | 10 |
|  |  |  |  |  |  |
|  |  |  |  |  |  |

**Table S3.** Regions exhibiting maximal connectivity to the motor cortex cluster, thresholded at Pearson´s r > 0.5. ^a^ Pearson´s r. ^b^ Coordinates are given in Talairach space
