# Supplementary material for: Platelet Serotonin Transporter Function Predicts Default-Mode Network Activity
Source: PLoS One. 2014 Mar 25;9(3):e92543. doi: 10.1371/journal.pone.0092543 (PMC3965432; doi:10.1371/journal.pone.0092543)
Supplement: Table S4 — Clusters of locally significantly increased serotonin transporter (5-HTT) availability compared to the whole cingulate cortex (CC). aUncorrected z-scores next to corresponding FWE-corrected one-tailed p values. bCoordinates are given in Talairach space. (DOC) [file pone.0092543.s013.doc]

| Cluster size | Region | Subregion | t | z^a^ | p | x^b^ | y | z |
| --- | --- | --- | --- | --- | --- | --- | --- | --- |
| 9 | aMCC | BA24 | 15.02 | 4.83 | 0.005 | 0 | 15 | 27 |
|  |  |  |  |  |  |  |  |  |
| 31 | sACC | BA32 | 14.83 | 4.81 | 0.005 | 2 | 31 | -8 |
|  | sACC | BA25 | 12.58 | 4.58 | 0.016 | 0 | 26 | -15 |
|  |  |  |  |  |  |  |  |  |
| 11 | pACC | BA32 | 13.32 | 4.66 | 0.011 | 2 | 39 | 0 |
|  |  |  |  |  |  |  |  |  |
| 3 | pMCC | BA24 | 12.56 | 4.58 | 0.016 | 6 | -9 | 47 |

**Table S4.** Clusters of locally significantly increased serotonin transporter (5-HTT) availability compared to the whole cingulate cortex (CC). ^a^ Uncorrected z-scores next to corresponding FWE-corrected one-tailed p values. ^b^ Coordinates are given in Talairach space.
