# Supplementary material for: Platelet Serotonin Transporter Function Predicts Default-Mode Network Activity
Source: PLoS One. 2014 Mar 25;9(3):e92543. doi: 10.1371/journal.pone.0092543 (PMC3965432; doi:10.1371/journal.pone.0092543)
Supplement: Text S1 — Supplementary methods. (DOC) [file pone.0092543.s014.doc]

**Supplementary Methods for**

Platelet Serotonin Transporter Function Predicts Default-mode Network Activity

**Task-related decreases and increases in activity**

Brain activations elicited by the task are shown in Fig. S1 and correspond to prior investigations applying negatively-valanced facial expressions and social stimuli . Activations within a widely-distributed neural network encompassing primarily areas of the medial temporal lobe including the amygdalae and the posterior cingulate cortex, wide parts of the secondary and tertiary visual cortex and parts of the frontal lobe have been found (Fig. S1). Moreover, significant deactivations have been detected in areas including the anterior cingulate cortex, the insula, left-hemispheric motor areas such as the precentral gyrus, subcortical areas such as the caudate and and parts of the parietal cortex.

**Cortical expression analysis of *SLC6A4***

The Allen Human Brain Atlas ([human.brain-map.org](http://www.human.brain-map.org/)) is a publicly available unique multimodal atlas of the human brain that integrates anatomic and genomic information . With respect to our study, we extracted *SLC6A4* microarray expression data for 37 cortical regions available for multiple adult control brain probes of European ancestry. We extracted expression data that has been provided as z-scores. Since up to eleven different probes are available for each brain region, we averaged all z-scores for identical regions. Mean cortical *SLC6A4* expression data have been ranked and displayed as polar chart using Excel software (Fig. S8). It is noteworthy that all core regions of the DMN system comprising the medial prefrontal cortex, posterior cingulate, precuneus, medial temporal and inferior parietal lobe show increased cortical 5-HTT expression compared to average 5-HTT expression in the brain.

**5-HTT availability within the cingulate cortex**

*Subjects*. Eight healthy male subjects (mean age 28±3.6) were included.

*PET-Imaging*. PET scans were performed at the Department of Nuclear Medicine of the MUV. [^11^C]DASB (3-amino-4-[N-methyl-N-[^11^C]methyl-amino-methylphenylsulfanyl]-benzonitrile) was synthesized at the Department of Nuclear Medicine of the MUV, Vienna, Austria and was prepared in a fully-automated C^11^‑methylation synthesizer (GE Medical Systems, Uppsala, Sweden). An amount of 2-9 GBq of [^11^C]DASB was prepared within 35±3 min. Subsequently, quality control was assessed measuring radiochemical and chemical purity (using analytical HPLC), pH, isotonicity, radionuclidic purity and residual solvents (using gas chromatography). Sterility and endotoxines were controlled after the release of the product. The injected radioactivity (mean=351 MBq, SD=60) was of high radiochemical purity (>95%, mean 97.3%) and of high specific activity (mean=47.5 GBq/µmol) at the time of injection. Dynamic PET scans were quantified using the “multilinear reference tissue model 2” (MRTM2) . The cerebellum was applied as reference region. All calculations were performed in PMOD image analysis software, version 2.95 (PMOD Technologies Ltd, Zürich, Switzerland, www.pmod.com). The binding potential represents a proportional relationship between B_max_ (binding site density) and K_d_ (dissociation constant, which is inversely associated to affinity) . All computed binding potential values of this study are BP_ND_ values .

*Analysis of regional 5-HTT availability.* Voxel-wise 5-HTT binding potential maps have been sensitized for the detection of local increases of 5-HTT availability within the CC, and have been normalized by dividing voxel values of 5-HTT availability through mean availability within the CC for each single subject. A t-test was performed in order to detect significant local increases of 5-HTT availability within the CC and results corrected for multiple comparisons (one tailed, p<0.05, Family Wise Error (FWE) corrected).

*Results.* Analysis revealed a significant increase of 5-HTT availability within the ACC as well as the anterior part of the midcingulate cortex when compared to average 5-HTT-binding within the CC (Fig.S9 and Table S4).

**References**

1. Haxby JV, Hoffman EA, Gobbini MI (2002) Human neural systems for face recognition and social communication. Biol Psychiatry 51: 59-67.

2. Vuilleumier P, Pourtois G (2007) Distributed and interactive brain mechanisms during emotion face perception: evidence from functional neuroimaging. Neuropsychologia 45: 174-194.

3. Sabatinelli D, Fortune EE, Li Q, Siddiqui A, Krafft C, et al. (2011) Emotional perception: meta-analyses of face and natural scene processing. Neuroimage 54: 2524-2533.

4. Jones AR, Overly CC, Sunkin SM (2009) The Allen Brain Atlas: 5 years and beyond. Nat Rev Neurosci 10: 821-828.

5. Haeusler D, Mien LK, Nics L, Ungersboeck J, Philippe C, et al. (2009) Simple and rapid preparation of [(11)C]DASB with high quality and reliability for routine applications. Appl Radiat Isot 67: 1654-1660.

6. Ichise M, Liow JS, Lu JQ, Takano A, Model K, et al. (2003) Linearized reference tissue parametric imaging methods: application to [11C]DASB positron emission tomography studies of the serotonin transporter in human brain. J Cereb Blood Flow Metab 23: 1096-1112.

7. Mintun MA, Raichle ME, Kilbourn MR, Wooten GF, Welch MJ (1984) A quantitative model for the in vivo assessment of drug binding sites with positron emission tomography. Ann Neurol 15: 217-227.

8. Innis RB, Cunningham VJ, Delforge J, Fujita M, Gjedde A, et al. (2007) Consensus nomenclature for in vivo imaging of reversibly binding radioligands. J Cereb Blood Flow Metab 27: 1533-1539.
